# Supplementary material for: Child food poverty prevalence and its associated factors in Dale district, Sidama region, Ethiopia
Source: PLoS One. 2025 Jul 16;20(7):e0326999. doi: 10.1371/journal.pone.0326999 (PMC12266419; doi:10.1371/journal.pone.0326999)
Supplement: Attachment — Submitted filename: Point by point Response to reviwers.docx [file pone.0326999.s003.docx]

**Point by Point Response to Editorial and reviewers’ comments (manuscript Id: PONE-D-25-13161)**

For their helpful, constructive criticism and recommendations on how to make this manuscript better, we are grateful to the reviewer or reviewers. As indicated by the red colors, we, the authors, tried to understand and consider every suggestion and comment made in the main document. The entire manuscript has also been edited for grammar and English usage. The online system has the final, edited manuscript uploaded. We review your helpful, insightful response and recommendation.

| Comments category | Comments given | Point-by-point Amendments |
| --- | --- | --- |
| Reviewer #1: | | |
| The authors did an impressive work of outlining and explaining the full details of their work. I have few points for authors to reconsider and edit:  1. In the first paragraph of the introduction, you stated "The main causes of this illness include...." Child food poverty is NOT an illness, but it can lead to sever illnesses | | Dear reviewer, thank you for your detailed observation and it has been corrected and highlighted in the main revised manuscript. |
| 2. In the Second paragraph of the introduction, you mentioned the meaning of UNICEF acronym. however, this is not the full correct meaning | | It has been corrected to “United Nations International Children’s Emergency Fund (UNICEF)” |
| 3. In Materials and methods, Study area, you mentioned kebeles. This word is specific to Ethiopia only and not well known around the world. Therefore, it is preferable to mention what the meaning of this word is first and then you can use it freely. | | Dear reviewer, thank you for your detailed observation and it has been corrected and highlighted in the main revised manuscript. |
| 4. In Materials and methods, Study area, second paragraph, the author mentioned Ensete ventricosum and Catha edulis. Please make sure they are written in italic (scientific names of plants) | | It has been addressed based on your valuable comments. |
| 5. Figure 1 should show the highlighted kebeles where the investigation took place | | Dear reviewer, it is clearly stated in the revised manuscript. |
| 6. In Sample size determination, Sample size for first objective, state the meaning of α and p | |  |
| 7. In Variables of study, authors mentioned ANC and PNC for the first time here without explaining the meaning of Acronyms. Please state their meaning | | Dear reviewer, thank you and state. |
| 8. In Food insecure, HH was used to refer to household (maybe!!?). Please make it clear | | Dear reviewer, thank you for your detailed observation and have been corrected. |
| 9. In Data quality control, authors stated "Additionally, the supervisors will receive training on how to evaluate the consistency and thoroughness of surveys"will receive is not appropriate here | |  |
| 10. In Data quality control, VIF was used without explaining its meaning | |  |
| 11. In table 4, please state the meaning of ETB here | | Dear reviewer, thank you for your detailed and valuable observation that has been revised and replaced by $. |
| 12. In Table 5, authors regarded parents AND family members as separate sources of information. Why are parents and family members not in the same category? Do all pregnant women in this study have Family members besides parents? | | Dear reviewer, thank you for your insightful comments and accepted and corrected them based on your valuable comments. |
| 13. In figure 3, authors used acronyms without prior explanation of their meaning in the previous paragraph | | It has been mentioned and highlighted in the revised manuscript. |
| 14. In the second page of the discussion, authors mentioned IYCF, MDD without prior explanation of their meaning in the previous paragraphs | | Dear reviewer, thank you for your critical observation and clearly mentioning their meaning. |
| 15. In the second page of the discussion, authors should avoid using " in our nation" | | Dear reviewer, your valuable suggestion was accepted and removed. |
| 16. In the third page of the discussion, authors need to clarify their statement. This study demonstrated that being a female is associated with child food poverty. THIS IS THE FIRST LINE This could be attributable to male patriotism (FAUVORISISM) instead of patriotism in some communities where girls are less socially and nutritionally favored. This study was in line with studies conducted in Ethiopia such as Mekelle City [9], Tehuledere district(54), Jimma (55), Chiro town(56), and Wukro town (57) which confirmed that males were more affected due to thinness than girls. This variable includes several African countries including Pakitan (58). THE END OF THE PARAGRAPH DOES NOT AGREE WITH THE FIRST LINE THIS PARAGRAPH CONTRADICT ITSELF because it mentioned that male ate more affected than female even though it stated different thing in the first line. | | Dear reviewer , we the authors thank you so much and your cretical obsevation was adreessed at the revised manuscript and highlighted by red color. |
| 17. Authors mentioned a section called Abbreviation and or acronyms. Preferably, authors should state the meaning of their Abbreviation and or acronyms on their first appearance or mention in the manuscript to make the flow of the paper more easy to follow for the reader | | Dear reviewer , thank you for your detailed and valuable constructive comments. |
| 18. In the reference section, authors referred to websites. In this case, authors should include the website url, and date of obtaining the data Author should improve their manuscript by using English-editing service to avoid grammatical errors, etc.... | |  |
| **Reviewer #2:** | | |
| This is a commendable and timely study addressing child food poverty in a high-need region of Ethiopia. This study provides important insights into a critical area of public health nutrition. The identification of significant predictors—such as child age, sex, ANC follow-up, family size, and maternal knowledge—provides actionable insights for programmatic interventions. The discussion is well-supported with relevant literature and highlights practical implications. Regarding the technicality of the manuscript, the manuscript is technically sound and presents a well-executed community-based cross-sectional study involving 503 participants. The research rigorously followed scientific procedures, and the results logically supported the conclusions. Statistical analysis is thorough and appropriate for a cross-sectional study. Also, the use of standardized indicators, validated tools, and rigorous statistical analysis adds credibility to the findings. These methods are robust and validate the associations claimed in the study. The manuscript is well-written and easy to follow. The abstract, introduction, methods, results, and discussion are all structured logically and adhere to standard academic English. | | Dear reviewer, I thank you so much for your valuable and motivational detailed observation and we the authors tried to address as follows your insightful comments. |
| 2) Although ethical approval is mentioned, the Ethics Statement in the manuscript should be expanded. Explicitly mention:  a) Consent procedures (written/oral)  b) Ethical review body (Yirgalem Hospital Medical College)  c) Confidentiality measures (specify whether data were anonymized before analysis). | | Dear reviewer, we the authors have addressed this explicitly based on your valuable suggestion in the main revised manuscript. |
| 3) On Data Availability and Consent Statements, the manuscript says, “Data are available from the corresponding author upon request”, which contradicts PLOS ONE’s open data policy. Instead, the data should be made publicly available or archived in a repository, with links included. | | Thank you for your insightful comments accepted with modification of the revised main manuscript and higlighted with red color. |
| 4) The introduction provides a solid background, but the review can be improved by integrating more recent global data on child food poverty and nutrition transitions | | Dear reviewer, thank you for your insightful comments, which have been addressed and highlighted in the revised manuscript. |
| 5) While the introduction touches on the knowledge gap, the authors should emphasize more clearly what this study adds that others have not done in Sidama or Ethiopia more broadly. | |  |
| 6) Recommendations should be more action-oriented. Rather than general calls to “raise awareness,” suggest specific programs or stakeholder groups. | | Dear reviewer, it has been corrected based on your valuable suggestion in the revised manuscript. |
| 7) There are occasional typos that should be corrected during revision, and they include:  a) foodv poverty in the conclusion rather than food poverty.  b) Abbreviations not abbrivation  c) ANC: Antenatal Care not Ante natal Care  d) AOR: Adjusted Odds Ratio not Adjust Odd Ratio  e) EBF: Exclusive Breastfeeding not E xclusive Breastfeeding (excessive spacing)  f) EDHS: Ethiopian Demographic and Health Survey not Ethiopian Demography and Health Survey  g) IDD: Inadequate Dietary Diversity not Iinadequte Dietary Diversity (double “I”)  h) MDGs: Millennium Development Goals not Millennium Development Goals’ (wrong apostrophe)  i) PNC: Postnatal Care not Post Natal Care  j) SPSS: Statistical Package for the Social Sciences not Statistical Package for the Social Science | | Dear reviewer, I thank you so much for your detailed observation and it has been corrected in the revised manuscript based on your valuable observation. |
| 8) I would recommend that acronyms be presented in alphabetical order for better readability. | | Dear reviewer, it has been accepted and highlighted in the revised manuscript. |
| 9) A consistent colon and space format should be used especially in the abbreviation and/or acronym section as well as throughout the body of the article. | |  |
| 10) Use "to" instead of a dash for formal writing (February 15 to March 15, 2024) at the Study design and period. | |  |
| **Reviewer 3** | | |
| 1. English language editing is needed in some parts of the study. | | Dear reviewer, of course, and edited. |
| 2. The title is informative but too long and wordy. I suggest the following title: [Prevalence and Determinants of Child Food Poverty in Dale District, Sidama Region, Ethiopia]. | | Dear reviewer, your suggestion has been accepted and modified as “Child food poverty prevalence and its associated factors in Dale district, Sidama region, Ethiopia” |
| 3. Terminologies like “child food poverty” and “inadequate feeding” are used interchangeably, which may confuse readers. Define each key term clearly and consistently stick to one throughout the paper, especially in the abstract and introduction. | | Dear reviewer, thank you for your detailed observation and clearly stated in the operational definition and used throughout the revised manuscript. |
| 4. In Introduction: The study aim is broad and not broken down into measurable components. Try using the term (prevalence). | | Dear reviewer, thank you for your valuable critical comments and the title modification. |
| 5. In Methods: The study period (February 15 – March 15, 2024) is listed, but seasonal variation that affects food availability is not discussed. Mention why this time frame was chosen and how it might affect feeding practices due to the seasonal availability of food. | | Dear reviewer, it has been stated clearly and highlighted with the red color as you suggested. |
| 6. In Methods: The map and sampling diagram (Figure 1 and Figure 2) are mentioned, but not included in the provided text, and there's no description of what they show. Ensure all figures are labeled, included, and properly referenced in-text with brief captions. | | Dear reviewer, thank you for your critical and constructive feedback. It has been addressed and highlighted in the revised manuscript. |
| 7. In Methods: The sampling method may not be well explained. How were households selected? Were clusters used? Any stratification? Please respond clearly. | |  |
| 8. In limitations of the study: Limitations related to cross-sectional design, and generalizability are not addressed. Mention that causality cannot be inferred due to the study design. | |  |

Fentaw Wassie Feleke: [fentawwassie@gmail.com](mailto:fentawwassie@gmail.com) (on behalf of all the authors). Thank you in advance!
